# Improved remnant polarization of Zr-doped HfO_2_ ferroelectric film by CF_4_/O_2_ plasma passivation

**DOI:** 10.1038/s41598-022-21263-8

**Published:** 2022-10-06

**Authors:** Yejoo Choi, Hyeonjung Park, Changwoo Han, Jinhong Min, Changhwan Shin

**Affiliations:** 1 Department of Electrical and Computer Engineering, Sungkyunkwan University, Suwon, 16419 South Korea; 2 Department of Materials Science and Engineering, University of Michigan, Ann Arbor, MI 48109 USA; 3 School of Electrical Engineering, Korea University, Seoul, 02841 South Korea

**Keywords:** Engineering, Nanoscience and technology

## Abstract

In this work, the impact of fluorine (CF_4_) and oxygen (O_2_) plasma passivation on HfZrO_x_ (HZO) based ferroelectric capacitor was investigated. By the fluorine passivation, the surface trap density and oxygen vacancies in the HZO-based Metal–ferroelectric–insulator–semiconductor (MFIS) capacitors were suppressed, resulting in the increased pristine remnant polarization (2P_r_). The pristine value (2P_r_) of baseline samples annealed at 500 °C and 600 °C were 11.4 µC/cm^2^ and 24.4 µC/cm^2^, respectively. However, with the F–passivation, the 2P_r_ values were increased to 30.8 µC/cm^2^ and 48.2 µC/cm^2^ for 500 °C and 600 °C, respectively. The amount of surface defects and oxygen vacancies are quantitatively confirmed by the conductance method and XPS analysis. However, due to the incorporation of fluorine atoms into the ferroelectric–insulator films, undesirable degradation on endurance characteristics were observed.

## Introduction

Since the ferroelectricity of HfO_2_ was discovered in 2011, HfO_2_-based ferroelectric field effect transistors (FeFETs) have attracted a lot of interest for future nonvolatile memory applications because of their compatibility to complementary metal-oxide-semiconductor (CMOS) technology as well as superior scalability^1–3^. However, poor surface quality of ferroelectric layer degrades the device performance. Defects on the surface of ferroelectric materials would increase the depolarization field in the material, and/or they would create a dead layer (i.e., not too much polarization would be induced in the thin “dead” layer of ferroelectric materials). This causes the remnant polarization (2P_r_) of ferroelectric materials to be degraded^4,5^. In addition, bulk defects of ferroelectric materials (herein, ferroelectric HfO_2_), which are mostly consisted of oxygen vacancies, would be also responsible for 2P_r_ degradation^6^. The oxygen vacancies are expected to have strong impacts on the ferroelectricity including remnant polarization and endurance performance^7,8^. Because the oxygen vacancies induce the formation of the non-ferroelectric dead layer in the interface. This causes the polarization domain pinning effect, resulting in pinched polarization- vs. -voltage (P–V) characteristics and it leads to the degradation of reliability properties^7^. In the previous study, it turned out that fluorine plasma treatment can passivate surface/bulk defects in Al-doped HfO_2_ ferroelectric film^9^. However, the fluorine plasma treatment on HfO_2_ dielectric film would cause excessive incorporation of fluorine atoms to Hf/Zr atoms in HZO, resulting in the formation of interlayer (IL) (and therefore, degraded dielectric constant^10^.

While CF_4_/O_2_ plasma treatments for passivation of surface/bulk defects were used for various types of thin films, study on the impact of CF_4_ and O_2_ passivation on ferroelectric film is still lacking. In this work, the effects of CF_4_ and O_2_ plasma passivation on the remnant polarization and endurance characteristics of ferroelectric-based MFIS capacitor are investigated. The quantitative analysis such as XPS and conductance method has been done to analyze the quantity of surface defects and oxygen vacancy in ferroelectric films. Furthermore, poor endurance characteristics of ferroelectric film (which is caused by excessive incorporation of fluorine atoms to Hf/Zr atoms) were first observed.

## Materials and methods

MFIS (metal/ferroelectric/insulator/semiconductor) capacitors were fabricated on 150 mm silicon wafer. First, standard cleaning works and diluted HF (1:50) cleaning works were done for p-Si (100) wafers with resistivity of < 0.005 Ω∙cm. Afterwards, 1-nm-thick SiO2 was formed by wet chemical oxidation using a HPM (HCl:H_2_O2:H_2_O = 1:1:5). Then, a 10-nm-thick HZO (Zr-doped HfO_2_) was deposited by thermal atomic layer deposition (ALD), in which tetrakis (ethymethylamino) hafnium (TEMAH) precursor, tetrakis (ethylmethylamino) zirconium (TEMAZ) precursor, and H_2_O source were used. In order to passivate surface defects and oxygen vacancies, fluorine plasma passivation (F-passivation) was done by chemical dry etcher (CDE). Note that three different conditions for the fluorine plasma treatment were used, i.e., baseline (no F-passivation), CDE1, CDE2, and CDE3. In detail, the O2 gas flow rate of CDE1, CDE2, and CDE3 were 30 sccm, 40 sccm, and 60 sccm, respectively. All the other conditions such as CF_4_ gas flow rate were identical for all the samples. After the F-passivation, 50-nm-thick TiN was deposited by physical vapor deposition (PVD), followed by post-metallization annealing (PMA) for the crystallization of HZO film (note that the PMA was applied, as well, for the baseline sample). To explore the impact of the annealing temperature on baseline/CDE1/CDE2/CDE3, three different temperatures, i.e., 500 ℃, 600 ℃, and 700 ℃, were used for 30 s in N_2_ atmosphere.

The device measurements were done with Keithley 4200A-SCS parameter analyzer, to characterize the ferroelectric properties of those MFIS capacitors. The capacitance versus voltage (C–V), endurance characteristics were measured. A triangular waveform with the amplitude of 4 V was used to characterize the polarization–voltage (P–V) characteristics. A trapezoidal waveform with the amplitude of 4 V was used for the endurance cycling. Note that the rise/fall time for both waveforms and the pulse width for trapezoidal waveform were set to 1 μs.

## Results and discussion

**Figure 1:**
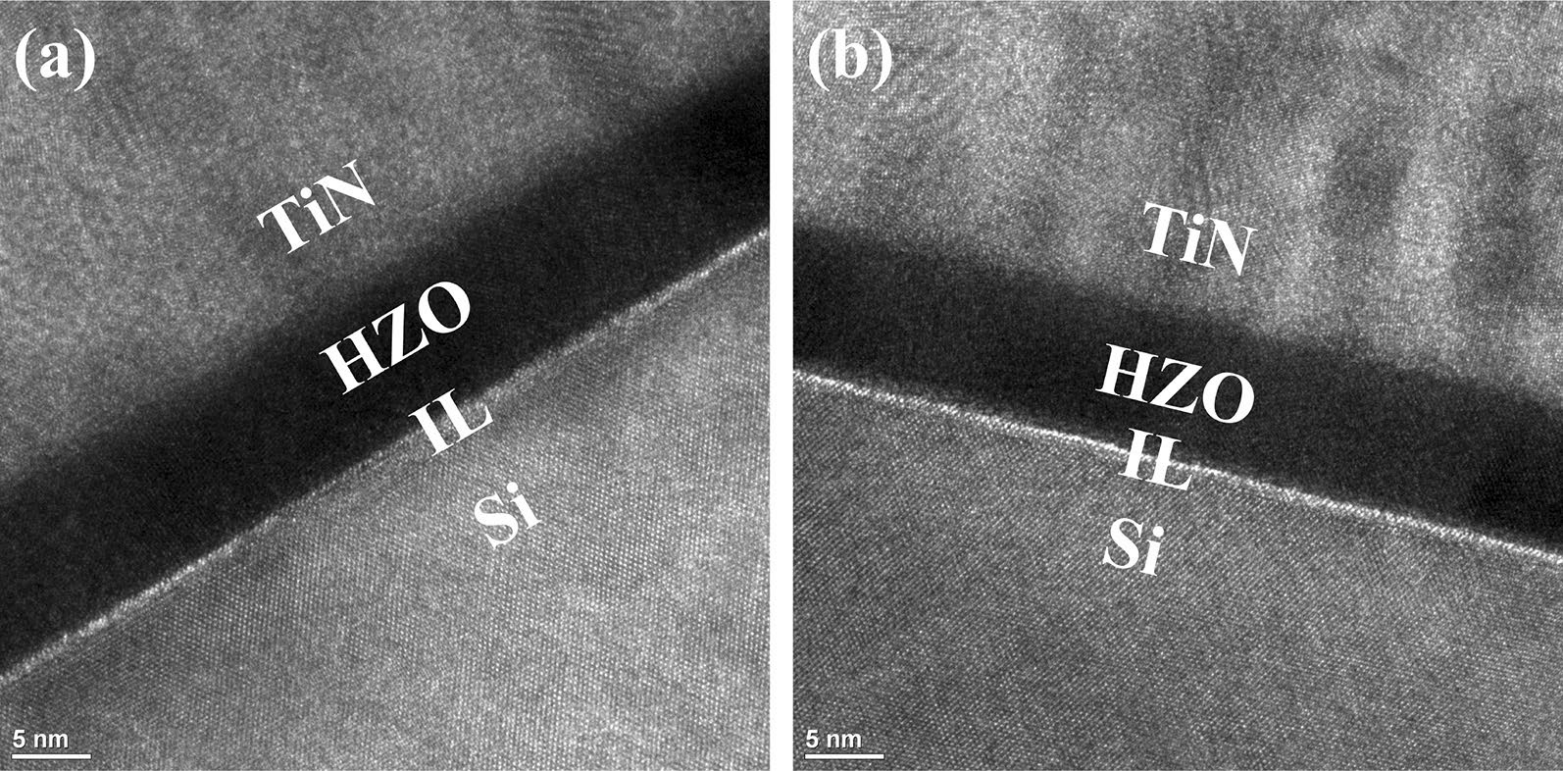
TEM image of (**a**) baseline MFIS capacitor and (**b**) MFIS capacitor treated by fluorine plasma. Note that the IL layer was slightly regrown in the F-passivation process.

**Figure 2:**
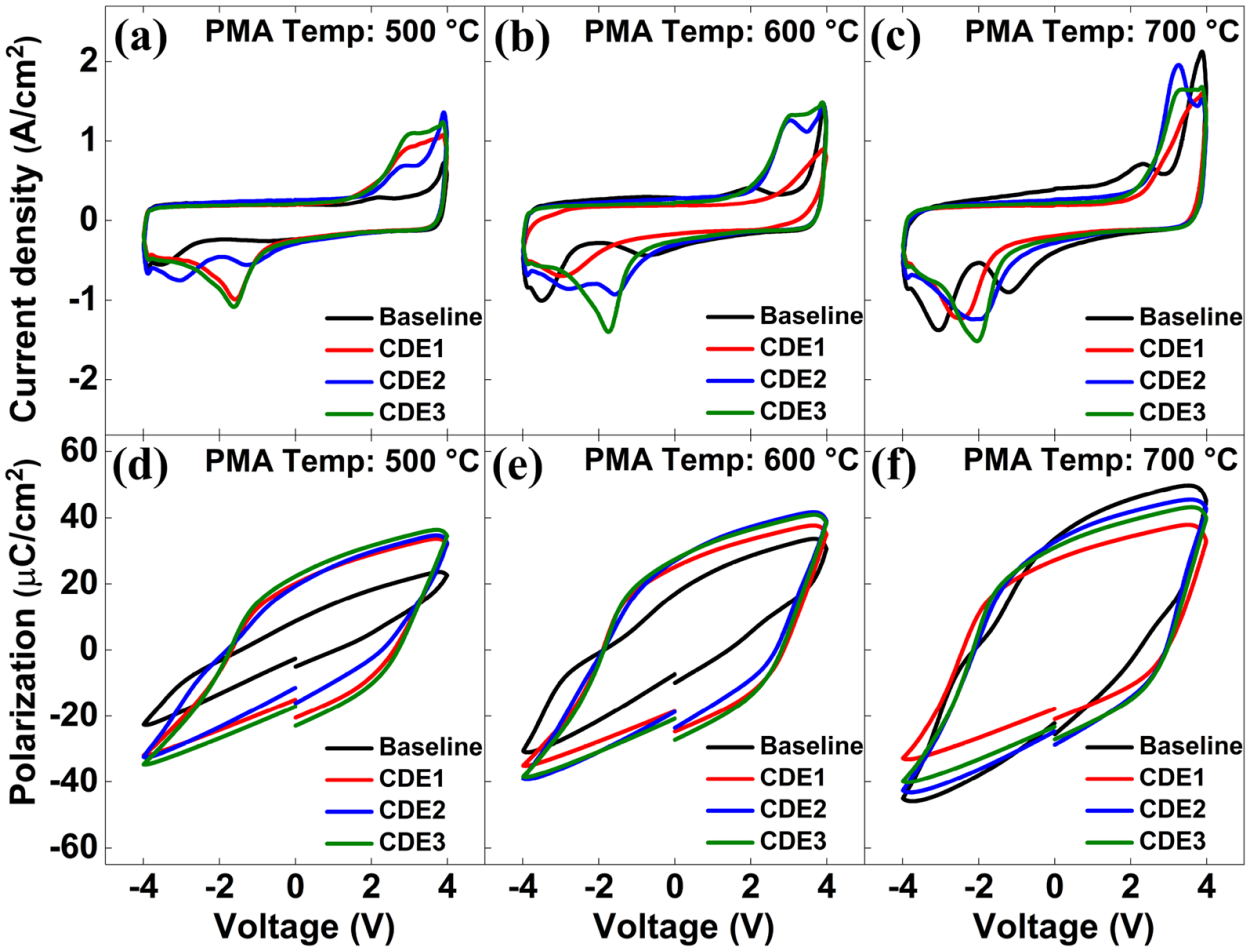
Measured current density versus voltage and pristine polarization versus voltage for three different post metal annealing (PMA) temperatures: (**a,d**) 500 °C, (**b,e**) 600 °C, and (**c,f**) 700 °C. Chemical Dry Etch (CDE) 1, 2, and 3 indicates the passivated samples (Baseline sample was not exposed to the passivation). The O_2_ plasma gas flow conditions for CDE1, CDE2, and CDE3 are 30 sccm, 40 sccm, and 60 sccm, respectively. Note that the fluorine plasma gas flow for all the samples was identically set to 80 sccm.

Figure 1 shows the TEM images of MFIS capacitor with/without fluorine plasma treatment. As shown in Fig. 1b, the treatment did not affect the physical thickness of HZO layer. Figure 2 shows the measured current- vs. -voltage (I–V) and pristine polarization- vs. -voltage (P–V) of MFIS capacitors with various post metal annealing (PMA) temperatures (T_A_). The pristine value of 2P_r_ of baseline samples were 11.4 µC/cm^2^ and 24.4 µC/cm^2^ at T_A_ of 500 ℃ and 600 ℃, respectively. However, with the help of the fluorine plasma treatment (i.e., F-passivation), the 2P_r_ values were increased to 30.8 µC/cm^2^ and 48.2 µC/cm^2^ at T_A_ of 500 ℃ and 600 ℃, respectively. This is because fluorine atoms can passivate both (i) surface defects of HZO film and (ii) oxygen vacancies in HZO film: It is known that surface defects would create a dead layer on a ferroelectric interface, which degrades the 2P_r_ value of the ferroelectric capacitor^4^. It is also known that nonuniformly distributed oxygen vacancies in HZO layer would cause internal electric field in the HZO film, which negatively affects the 2P_r_ value^6,11^. Surface defects on HZO layer can be measured by the conductance method: the Eq. (1) is for trap density (D_it_), and the Eq. (2) is for parallel conductance (G_p_)^2^.

1 $${D}_{it}=\frac{2.5}{Aq}{\left(\frac{{G}_{p}}{\omega }\right)}_{peak},$$

2 $$\frac{{G}_{p}}{\omega }=\frac{\omega {{C}_{ox}}^{2}{G}_{m}}{{{G}_{m}}^{2}+{\omega }^{2}{({C}_{ox}-{C}_{m})}^{2}}.$$

**Figure 3:**
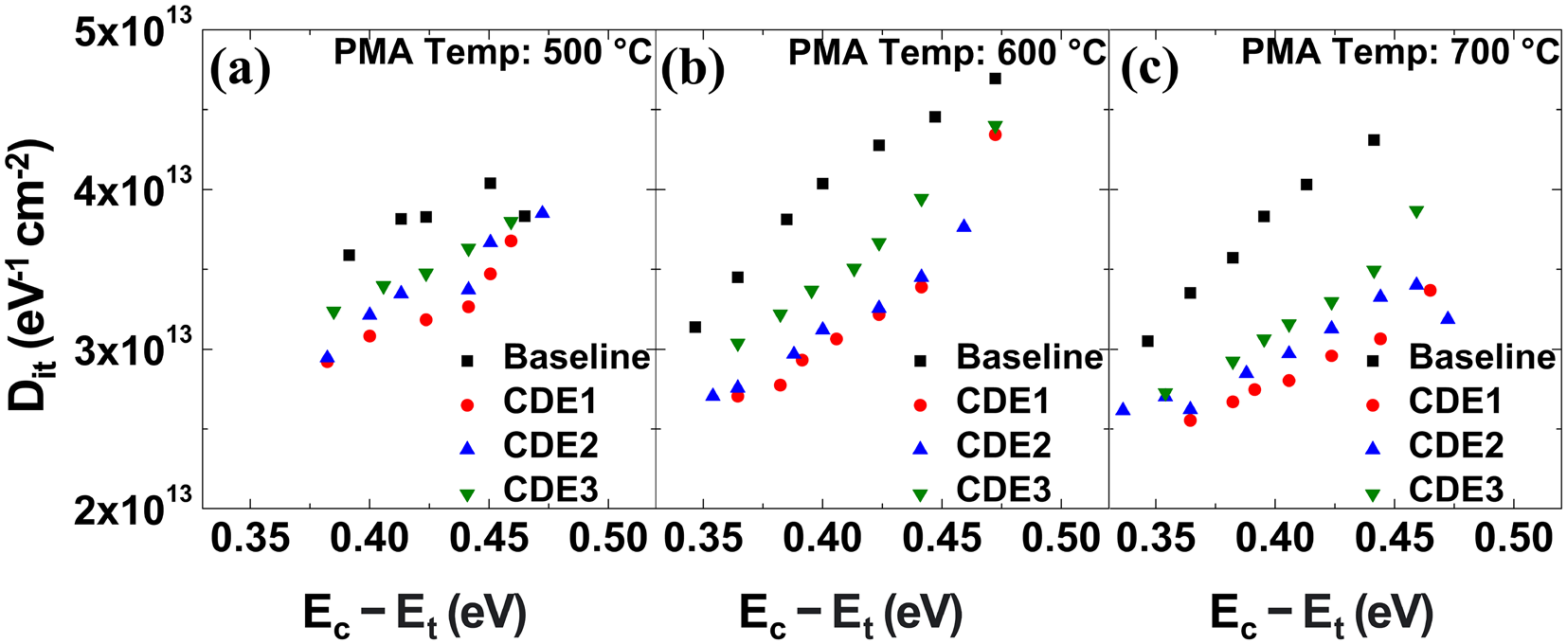
Trap density at the interface of SiO_2_/Si in MFIS capacitor for three different PMA temperatures: (**a**) 500 °C, (**b**) 600 °C, and (**c**) 700 °C. Note that the trap density was measured using the conductance method.

In conductance method, the equivalent circuit of MFIS capacitor consists of oxide capacitance (C_ox_), semiconductor capacitance (C_s_), interface trap capacitance (C_it_), and interface trap resistance (R_it_)^12^. Using the loss mechanism of capture and emission process (which is occurred due to the charge trapping at interface traps), the trap density can be extracted. The time constant of interface trap, i.e., *τ*_*it*_ = *R*_*it*_*C*_*it*_, can be obtained by measuring the conductance and capacitance of MFIS capacitor. In the Eqs. (1) and (2) above, notice that ω (= 2πf), A, q, G_m_, and C_m_ indicate angular frequency, capacitor area, unit charge, measured conductance, and capacitance, respectively. The oxide capacitance (C_ox_) in Eq. (2) is obtained as the measured capacitance of MFIS capacitor in strong accumulation mode^13^. As shown in Fig. 3, D_it_ of fluorine treated samples (i.e., CDE1, CDE2, CDE3) is much lower than that of non-treated one. This explicitly indicates that fluorine atoms have well passivated the surface defects on HZO layer.

In contrary to T_A_ of 500 ℃ and 600 ℃, it turned out that the pristine 2P_r_ value of HZO ferroelectric capacitor was not significantly improved at T_A_ of 700 ℃ (see Fig. 2c vs. Fig. 2a,b). The reason why it was hard to see the improvement of 2P_r_ at 700 ℃ is as follows: The traps (i.e., D_it_) in baseline MFIS capacitor at 700 ℃ (vs. 500 ℃ and 600 ℃) were already uniformly distributed due to the high annealing temperature (see Fig. 3c vs. Fig. 3a, b; see the baseline samples only)). The uniform distribution of defects leads to the decrease of built-in bias and results in the increase of remnant polarization in higher annealing temperature. The uniformly distributed traps (especially, oxygen vacancies) in HZO layer would not significantly cause the internal electric field in the film, so that the higher T_A_ (i.e., 700 ℃) would not significantly contribute to enhancing the pristine 2P_r_ value.

**Figure 4:**
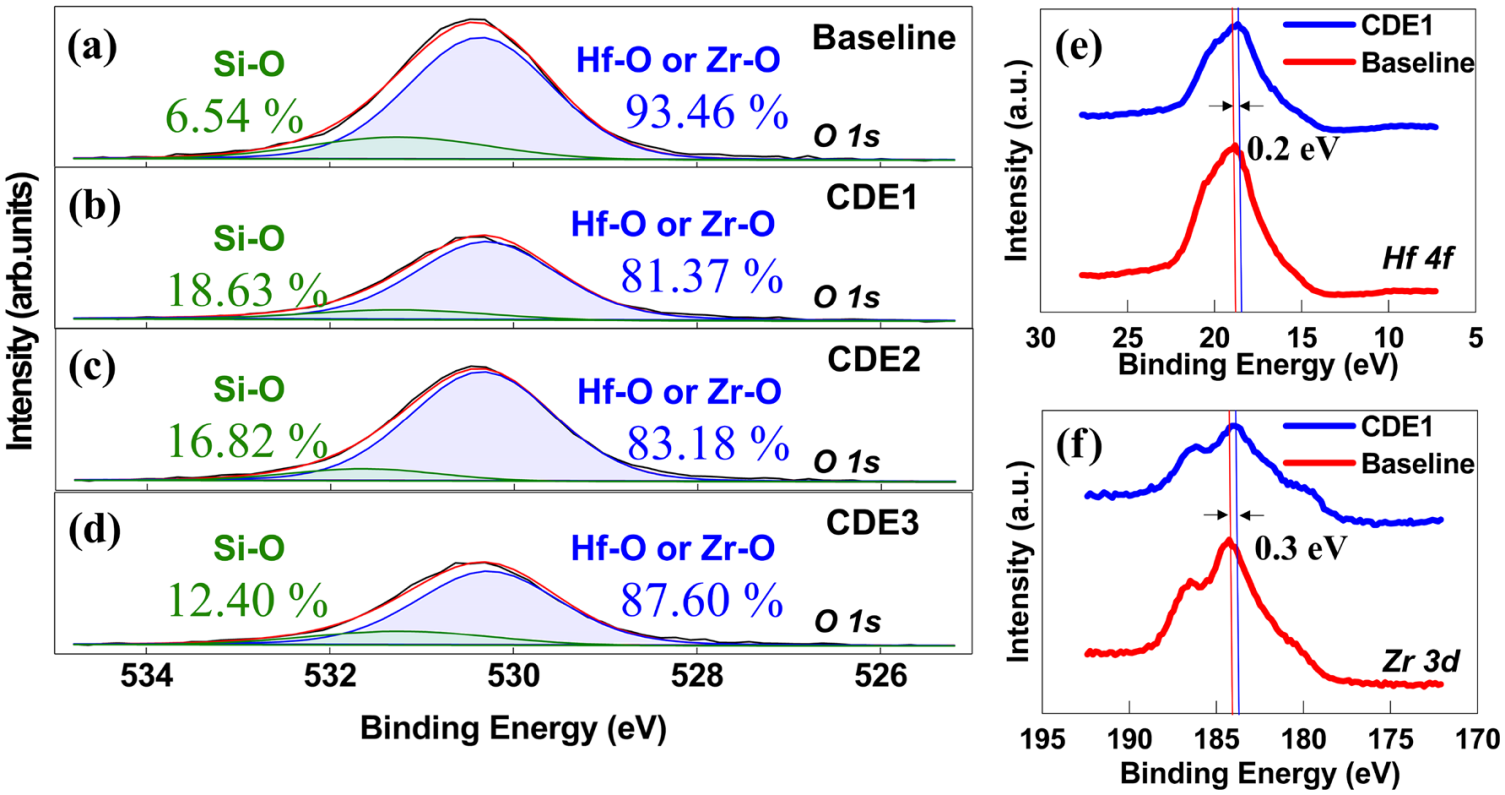
XPS spectra for (**a**) baseline, (**b**) CDE1, (**c**) CDE2, and (**d**) CDE3 MFIS capacitors. (**e**) Hf 4f XPS spectra for baseline and CDE1 sample, (**f**) Zr 3d XPS spectra for baseline and CDE1 sample.

During the chemical process of F-passivation, some of oxygen atoms are replaced with fluorine atoms^10^. In other words, some of Hf–O or Zr–O bonds are replaced with Hf–F or Zr–F bonds. Figure 4a–d confirms this with X-ray Photoelectron Spectroscopy (XPS) analysis for all passivation conditions at T_A_ of 600 ℃. The O 1s spectra can be deconvoluted into 2 peaks, where low band indicates Hf–O or Zr–O bonds, while high band indicates Si–O bonds^14,15^. This shows that fluorine-treated capacitors have more content of Si–O bonds than baseline capacitors. With the fluorine plasma treatment, the contents of Si–O bonds was increased up to 18.63%, 16.82%, and 12.40% for CDE1, CDE2, and CDE3, respectively (6.54% only in the baseline capacitor). This can be understood that a higher O_2_ contents in plasma treatment (herein, CDE3 has the highest O_2_ contents) induces a lower Si–O contents in MFIS capacitor.

Once some of Hf–O or Zr–O bonds are replaced with Hf–F or Zr–F bonds, oxygen atoms can be released out, and then diffused toward interlayer (IL) of SiO_2_ in MFIS capacitor. In the chemical process of F-passivation (note that O_2_ plasma gas was used in the passivation), it turned out that the regrowth of IL layer is occurred together with the passivation of defects and the replacement of bonds mentioned above^16^. In this work, the regrowth of IL layer was explicitly observed, as shown in TEM images of all the MFIS capacitors (see Fig. 1). In addition, since XPS O1s spectra indicates the different ratio of Hf–O/Zr–O and Si–O in CDE1 to CDE3 (Fig. 4b–d), it can be inferred that the thickness of IL can controlled by optimizing the CF_4_/O_2_ gas flow rate. Figure 4e,f show Hf 4f and Zr 3d XPS spectra for baseline and CDE1 capacitors. The Hf 4f and Zr 3d peaks of baseline samples are located at 18.82 eV and 184.26 eV, respectively. After fluorine plasma treatment, Hf 4f and Zr 3d peaks move to 18.66 eV and 183.94 eV. That is, after F-passivation, Hf 4f and Zr 3d peaks were decreased by 0.16 eV and 0.32 eV, which means reduction of oxygen vacancies. The peak shifting after the F-passivation indicates the reduction of oxygen vacancies in HZO film^16^. When oxygen vacancies are reduced in HZO films, binding energy shifts to lower value, because oxygen vacancies in HZO films have positive charges^16^. This shows that the atomic fluorine interstitial effectively passivates the oxygen vacancy in the HfO_2_^17^.

**Figure 5:**
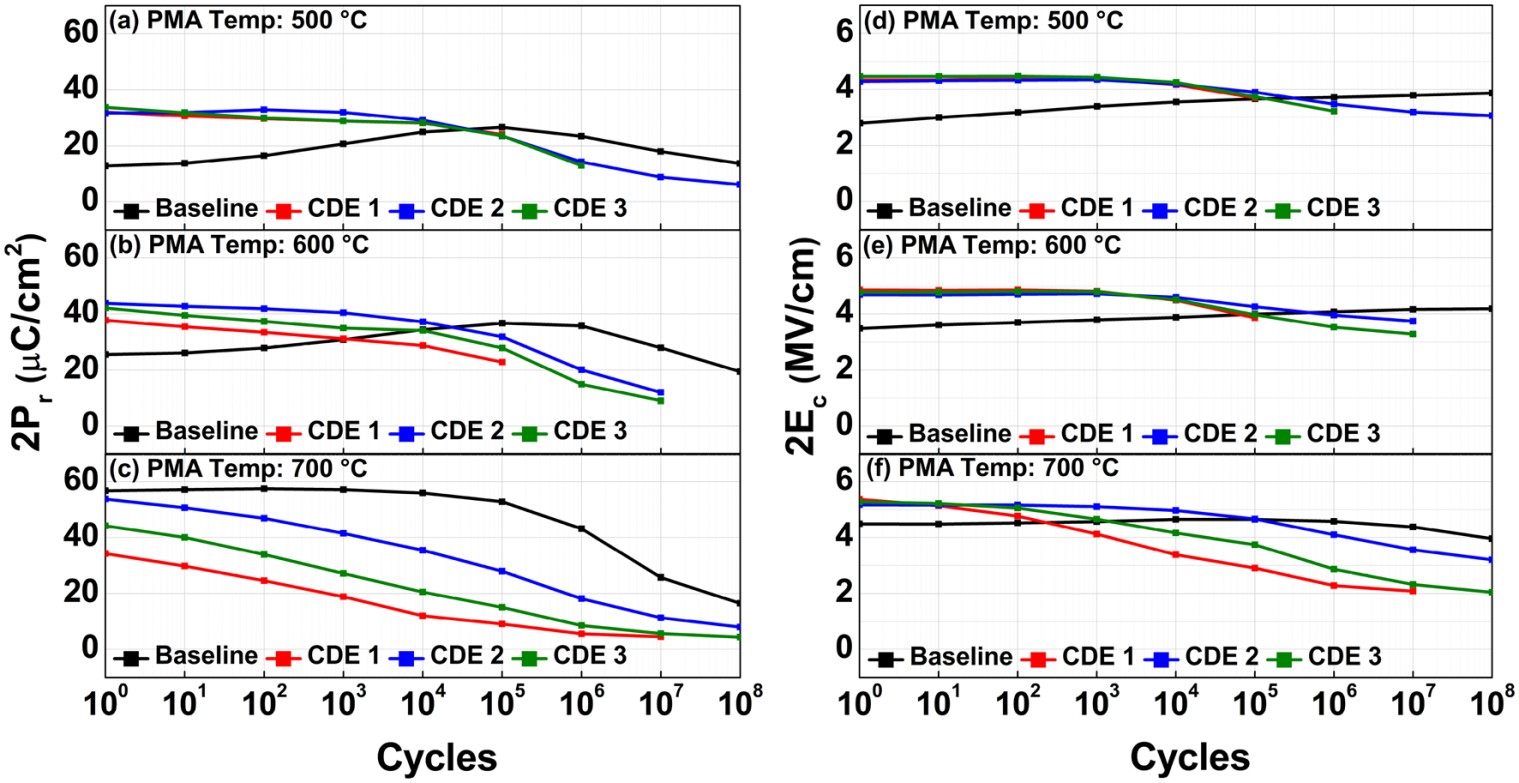
Measured 2P_r_ vs. the number of cycles and 2E_c_ vs. the number of cycles for endurance of the MFIS capacitors under three different PMA temperatures: (**a,d**) 500 °C, (**b,e**) 600 °C, and (**c,f**) 700 °C.

In Fig. 5, the measured endurance characteristics of each sample were exhibited. With increasing the number of cycles for P–V measurement, the CDE1/2/3 samples (vs. the baseline sample) showed no wake-up effect with the help of the passivation of surface defects and oxygen vacancies. Herein, the wake-up effect can be understood as increasing 2P_r_ with increasing the number of cycling. It has been known that the wake-up effect is originated from surface defects and oxygen vacancies in ferroelectric films. In a pristine state of ferroelectric films, locally distributed surface defects and oxygen vacancies would create a dead layer as well as the charges trapped in those defects/vacancies would occur an internal electric-field. The dead layer causes the polarization domain pinning effect, resulting in the pinched polarization- vs. -voltage (P–V) characteristics, and it leads to the degradation of reliability properties^7^. However, during the field cycling (i.e., in the process of repeatedly measuring P–V characteristics), surface defects and oxygen vacancies became uniformly redistributed, and thereby, the dead layer and internal electric-field can be suppressed^4,11^. It was revealed that temperature-dependent defects diffusion/drift were responsible for the wake-up and fatigue^11^.

**Figure 6:**
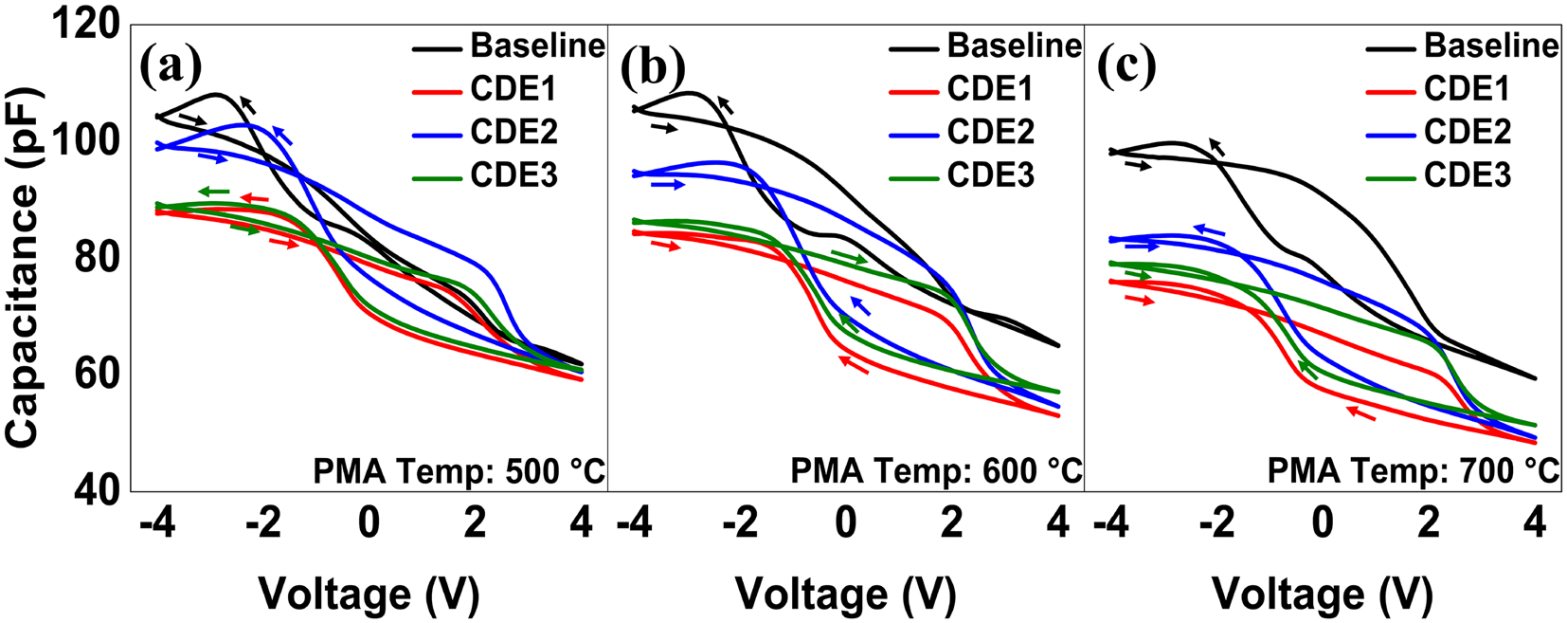
Measured capacitance- vs. -voltage of MFIS capacitors for three different PMA temperatures: (**a**) 500 °C, (**b**) 600 °C, and (**c**) 700 °C.

Although the wake-up effect was alleviated by fluorine plasma treatment, early fatigue and breakdown characteristics were not fully fixed. In the MIFS structure used in this work, its endurance characteristics are not limited by ferroelectric breakdown but by dielectric breakdown^18^. In real, it turned out that the dielectric IL in the MFIS structure underwent its regrowth in the fluorine plasma treatment, resulting in the reduced capacitance of the MFIS stack. This should induce a higher voltage (electric field) across the IL layer, and thereby, would cause the early dielectric breakdown^10,19,20^. The reduction of capacitance, i.e., an undesirable and secondary effect of F-passivation, was explicitly measured and shown in Fig. 6. The measured capacitance of MFIS capacitors (i.e., CDE1/2/3) were decreased at all T_A_. When fluorine atoms are incorporated into the MFIS stack, the dielectric constant of SiO_2_ was reduced due to those F atoms^10,20^.

## Conclusion

The effects of fluorine plasma passivation on HZO-based MFIS capacitor were investigated. Surface defects and oxygen vacancies on/in the HZO layer were well passivated by the fluorine plasma passivation, resulting in improved pristine 2P_r_ value. The decreased oxygen vacancies in HZO films were experimentally verified with the reduced binding energy of Hf 4f and Zr 3d on XPS spectra (i.e., Hf 4f and Zr 3d peaks were decreased by 0.16 eV and 0.32 eV). And the decreased trap density was quantitatively discussed and measured by the conductance method. Lastly, the endurance characteristics of HZO-based MFIS capacitor were studied. With increasing the number of cycles for P–V measurement, the CDE1/2/3 samples (vs. the baseline sample) showed no wake-up effect with the help of the passivation of surface defects and oxygen vacancies.

## Data Availability

The datasets generated during and/or analysed during the current study are available from the corresponding author on reasonable request.
